# Production, health‐promoting properties and characterization of bioactive peptides from cereal and legume grains

**DOI:** 10.1002/biof.1889

**Published:** 2022-09-26

**Authors:** Taiwo Ayodele Aderinola, Kwaku Gyebi Duodu

**Affiliations:** ^1^ Department of Food Science and Technology, School of Agriculture and Agricultural Technology The Federal University of Technology Akure Nigeria; ^2^ Department of Consumer and Food Sciences, Faculty of Natural and Agricultural Sciences University of Pretoria Hatfield South Africa

**Keywords:** anti‐inflammatory, antioxidant, bioactive peptides, cereal grains, legume grains

## Abstract

The search for bioactive components for the development of functional foods and nutraceuticals has received tremendous attention. This is due to the increasing awareness of their therapeutic potentials, such as antioxidant, anti‐inflammatory, antihypertensive, anti‐cancer properties, etc. Food proteins, well known for their nutritional importance and their roles in growth and development, are also sources of peptide sequences with bioactive properties and physiological implications. Cereal and legume grains are important staples that are processed and consumed in various forms worldwide. However, they have received little attention compared to other foods. This review therefore is geared towards surveying the literature for an appraisal of research conducted on bioactive peptides in cereal and legume grains in order to identify what the knowledge gaps are. Studies on bioactive peptides from cereal and legume grains are still quite limited when compared to other food items and most of the research already carried out have been done without identifying the sequence of the bioactive peptides. However, the reports on the antioxidative, anticancer/inflammatory, antihypertensive, antidiabetic properties show there is much prospect of obtaining potent bioactive peptides from cereal and legume grains which could be utilized in the development of functional foods and nutraceuticals.

## 1 INTRODUCTION

Protein is an essential dietary nutrient and one of the major macronutrients needed by the body for growth, development, regulation, and maintenance. In recent times however, more attention has been focused on the biologically active protein fragments—bioactive peptides (BAPs). BAPs are small proteins composed of short‐chain (<20) amino acid residues. They could be released from the inactive parent protein chemically or enzymatically (both endogenous and exogenous).^1^, ^2^ With increasing yearly publications of over 800 since 2014 in the area of BAPs,^3^ it is obvious that more research efforts are being put into this research field. This may be due to the gradual discovery and acknowledgment of the immense physiological roles of BAPs in the prevention, control and management of diseases.^4^, ^5^, ^6^

Again, this seems to indirectly indicate the subtle trend and change in consumer food choices. It is becoming more apparent than ever, that there is an increasing conscious demand for functional foods. That is, foods that are able to provide health benefits in addition to their normal supply of nutrients.^7^ In this regard, new food products are being developed which involve incorporation of isolated biologically active ingredients such as polyphenolic extracts and protein isolates, including hydrolysates and other peptide fractions for production of functional foods and nutraceuticals.

The functionality and utilization of BAPs have been greatly explored from various sources such as plants,^8^, ^9^, ^10^ microbes, animals, and their products (including fish).^11^, ^12^, ^13^, ^14^ Other high‐protein sources such as underutilized crops/seed and food wastes^15^, ^16^ have also been explored for BAP under various processing conditions because they are cheap and often easily available. Generally, protein functionalities are affected by the sequences and composition of its amino acid residues; consequently, the functionality and bioactivity of peptides are dependent on the sequences and composition of amino acid residues in the peptides.^17^ Various physiological functions such as antihypertensive, antidiabetic, immunomodulatory, antioxidant, and antimicrobial activities have been attributed to BAPs.^18^, ^19^, ^20^ Besides their utilization in the development of functional foods and nutraceutical, BAPs have also found relevance in other applications including wound healing and skin care products.^6^, ^21^

Cereal and legume grains form important parts of human diets worldwide and thus play an important nutritional role. While cereals are mostly starchy, legumes contain as much as twice the protein content (17%–40%)^22^ in cereals which ranges between 6% and 15%.^23^, ^24^ With respect to studies on BAP, cereal and legume grains appear to have been relatively under‐researched when compared to other food items. The aim of this review therefore, is to survey the current state of knowledge of BAPs from cereal and legume grains in terms of their production, health‐promoting properties, and characterization.

## 2 METHODS FOR PRODUCTION OF BIOACTIVE PEPTIDES FROM CEREALS AND LEGUMES

BAPs can be produced from foods through various methods. Basically, there are four commonly used methods for production of BAPs and these are enzymatic hydrolysis, in vitro gastrointestinal digestion, malting, and fermentation. These methods are hereby briefly discussed.

### 2.1 Production of bioactive peptides by enzymatic hydrolysis

Generally, an initial major step in the production of BAPs using this method is the isolation of the protein from the matrices of food after preliminary processing such as drying, milling, fat extraction, etc. depending on the nature of the food material. Several methods are available for protein extraction including aqueous extraction, iso‐electric precipitation, and alkaline solubilization among others. The isolates are subjected to hydrolysis, which are in most cases, carried out with enzymes either singly or in combination with other enzymes in what is called sequential or stepwise hydrolysis. Protein isolates from grains have been hydrolyzed with different enzymes such as trypsin, alcalase, pancreatin, pepsin, papain, etc.^17^, ^18^, ^25^, ^26^ As enzymes are highly specific, this implies different enzymes will produce hydrolysates or peptides with varied bioactivities. This is because the protein isolate peptide bonds are hydrolyzed at different locations, depending on the specificity of the enzymes thereby resulting in hydrolysates with different amino acid sequences.^7^ Previous studies on some grain protein isolates showed that hydrolyzing the same protein isolate with different proteases produced peptides with differing bioactivity. For instance, Agrawal et al.^17^ obtained higher antioxidant properties for the trypsin‐hydrolyzed finger millet for all the antioxidant assays when compared to the pepsin‐derived hydrolysate counterpart (Table 1). Similarly, pepsin‐derived pigeon pea hydrolysate had lower antioxidative capacity when compared to those hydrolyzed with pancreatin and pepsin‐pancreatin.^18^

**TABLE 1:** Antioxidative properties of cereal and legume grain protein and peptide fractions including their amino acid sequences

Source	Processing, purification, and characterization method	Test	Result	Sequence	Reference
Rice protein hydrolysate	Protein hydrolysis: Denazyme	H‐ORAC (l mol TE/g)	525.8	–	^27^
Brown rice	Pretreatment: Pasteurization at 121°C for 15 min	DPPH and ABTS (%)	DPPH Bromelain: 77 Protease FP51®: 53 ABTS Bromelain: 54 Protease FP51®: 37	–	^28^
	Protein hydrolysis: Bromelain and Protease FP51®				
Raw and boiled (10 min, 100°C, frozen yellow string beans (*Phaseolus vulgaris*)	Protein isolation: isoelectric precipitation—raw (PIR) and boiled (PIT)	MC (IC_50_ mg/ml), DPPH (%) and ABTS (%)	PIR MC: 0.42 DPPH: 23 ABTS: 54	–	^29^
			PIT MC: 0.33 DPPH: 32 ABTS: 51		
	Protein hydrolysis: Pepsin (PHR) and boiled (PHT)		PHR MC: 0.81 DPPH: 38 ABTS: 88		
			PHT MC: 0.19 DPPH: 46 ABTS: 92		
Corn germ	Protein hydrolysis: alcalase, flavourzyme and trypsin	DPPH, ABTS (μM TE/g dry matter), OH (μM histidine eq/g dry matter and MC (μM EDTA eq/g dry matter)	DPPH: 45 ABTS: 280 OH: 260 MC: 50	–	^30^
	Fractionation/purification Ultrafiltration (2, 10, and 100 kDa MWCO membranes) RP‐HPLC		DPPH: 38 (2–10 kDa) ABTS: 250 (<2 kDa) OH: 290 (2–10 kDa) MC: 47 (<2 kDa) DPPH: 65 (F1, trypsin) ABTS: 345 (F1, alcalase) OH: 470 (F1 trypsin) MC: 69 (F2, trypsin)		
Finger millet	Protein isolation: alkaline extraction	DPPH, ABTS, MC, OH (%)	DPPH: 25 ABTS: 27 MC: 23 OH: 27	TSSSLNMAVRGGLTR STTVGLGISMRSASVR	^17^
	Protein hydrolysis: trypsin and pepsin	DPPH, ABTS, MC, OH (%)	Trypsin DPPH: 38 ABTS: 40 MC: 35 OH: 33 Pepsin DPPH: 30 ABTS: 35 MC: 27 OH: 30		
	Fractionation/purification: Ultrafiltration (3 and 10 kDa) from trypsin hydrolysate Gel filtration RP‐UFLC Peptide identification: MALDI‐TOF/TOF‐MS/MS	DPPH, ABTS, MC, OH (<3 kDa) (%)	DPPH: 48 ABTS: 42 MC: 51 OH: 43 DPPH: 62 ABTS: 79 MC: 51 OH: 66 DPPH: 73		
Pearl millet	Protein isolation: Isoelectric precipitation	–	–	SDRDLLGPNNQYLPK	^31^
	Protein hydrolysis: Trypsin	DPPH, ABTS, MC, OH(%) and FRAP (absorbance)	DPPH: 26 ABTS: 26 MC: 23 OH: 30 FRAP: 0.269		
	Fractionation/purification RP‐UFLC		DPPH: 68 ABTS: 79 MC: 51 OH: 61 FRAP: 0.375		
Foxtail millet	Protein isolation: alcoholic (80% ethanol) extraction	–	–	–	^32^
	Protein hydrolysis: Alcalase (AH) and trypsin (TH)	DPPH (%) and ORAC (μM TE/g protein)	AH DPPH: 12 ORAC: 400 TH DPPH: 10 ORAC: 450		
	Fractionation/purification: Ultrafiltration (from alcalase: UFA, from trypsin: UFT) Gel filtration (from alcalase) RP‐HPLC		UFA (<1 kDa) DPPH: 17 ORAC: 650 UFT (<1 kDa) DPPH: 15 ORAC: 450 Gel fraction DPPH: 19 RP‐HPLC DPPH: 140		
White sorghum flour	Protein isolation: glacial acetic extraction	–	–	F3 fraction At 1.6 min KMVIV LAVCLA AVCLAL QQWQ QWQQ RQQCC MCGWQ CATSAAI At 3.6 min QWQQ QQWQ GVVQSV At 35 min QQWQ QWQQ GVVQSV QLQGVA VQQLQ VAQVAQ RQQCC MCGWVVQ CATSAAI DMQSR At 36 min KMVIV AVCLAL LAVCLA QQWQ QWQQ	^33^
	Protein hydrolysis: Alcalase (5 mg/ml sample concentration)	DPPH (%)	27		
	Fractionation/purification: Ultrafiltration: 1, 3, 5, and 10 kDa (10 mg/ml sample concentration)	DPPH (%), ABTS (%), FRAP (A_700_), MC (%)	DPPH: 78 (5–10 kDa) ABTS: 73 (<1 kDa) FRAP: 1.6 (3–5, 5–10, and > 10 kDa) MC: 23 (5–10 kDa)		
	Gel filtration chromatography (5–10 kDa) Sample concentrations DPPH: 5 mg/ml ABTS: 4 mg/ml Peptide Identification: MALDI‐TOF/TOF‐MS/MS	DPPH (%), ABTS (%), ORAC (g Trolox equivalent/g)	DPPH: 70 (F3) ABTS: 90 (F3) ORAC: 0.65 (F3)		
Lupin, quinoa, wheat	Protein hydrolysis: Solid state fermentation with three strains of *bifidobacteria* (*B. animalis*, *B. breve*, and *B. longum*) fermented for 0, 24, 48, and 72 h	DPPH and ABTS	DPPH Lupin: 61 (*B. longum*, 72 h) Quinoa: 65 (*B. longum* and *B. breve*, 24 h) Wheat: 37 (*B. breve*, 24 h) ABTS Lupin: 48 (*B. longum*, 72 h) Quinoa: 82 (*B. breve*, 24 h) Wheat: 36 (*B. animalis*, 0 h)	–	^34^
Amaranth	Protein isolation: Saline (albumin and globulin) and alkaline (glutelin) extractions	OH and ORAC	–	–	^35^
	Protein hydrolysis: *Lactobacillus helveticus*		Albumin OH: 27 ORAC: 1050 Globulin 11S OH: 49 ORAC: 600		
Pigeon pea	Protein isolation: Isoelectric precipitation (PpI)	DPPH, ABTS, OH (%) and FRAP (mmol Fe^2+^/mg)	DPPH PpI: 05 PpH: 34 PaH: 36 PpaH: 39 ABTS PpI: 18 PpH: 65 PaH: 75 PpaH: 72 OH PpI: 03 PpH: 09 PaH: 23 PpaH: 16 FRAP PpI: 0.01 PpH: 0.05 PaH: 0.07 PpaH: 0.04		^18^
	Protein hydrolysis: Pepsin (PpH), Pancreatin (PaH), pepsin‐pancreatin (PpaH)				

Abbreviations: ABTS, 2,2′‐azino‐bis(3‐ethylbenzothiazoline‐6‐sulfonic acid); DPPH, 2,2‐diphenyl‐1‐picrylhydrazyl; FRAP, ferric reducing antioxidant power; H‐ORAC, hydrophilic oxygen radical absorbance capacity; MALDI TOF/TOF MS, matrix‐assisted laser desorption/ionization time‐of‐flight/time‐of‐flight mass spectrometry; MC, metal ion chelation; OH, hydroxyl radical scavenging; RP‐UFLC, reverse phase ultra‐fast liquid chromatography.

### 2.2 Production of bioactive peptides by in vitro gastrointestinal digestion

In vitro gastrointestinal digestion (IGD) is a static digestion method simulating the natural digestion in the body. In order to ensure a more closely related digestion to that of the humans, which is dynamic, some modifications have been made to the method. These modifications include incorporation of different steps of digestion (mon‐, di‐, and multi‐step), addition of bile, consideration of transit and gastric emptying time, etc.^36^ A common model of the method, which is recognized internationally due to its simplicity, reproducibility and low cost assessment is the INFOGEST method.^36^, ^37^ The INFOGEST model is a three‐step method mimicking digestion at the oral, gastric, and intestinal (small intestine) phases as occurred in man. This method incorporates the main digestive enzymes at these three phases such as alpha amylase (oral), pepsin (gastric), and pancreatin (intestinal) as well as the bile salt. In study where there is a little or no starch, the oral phase may be removed.^38^ Moreover, the methods also artificially formulates and utilizes the various digestive fluids (as solvents for the digestive enzymes) at the three phases and these include: simulated salivary fluid (SSF, pH 7), simulated gastric fluid (SGF, pH 3), and simulated intestinal fluid (SIF, pH 7). As the sample is mixed with the digestive fluid, the pH and temperature of each simulated digestive fluid (a mixture of specific electrolyte solutions—potassium, sodium, magnesium, phosphate salts, bicarbonate, and calcium chloride) is adjusted and maintained at the optimum physiological pH and temperature (37°C). In most cases, digestion at the different phases lasts for 2, 120, and 120 min for SSF, SGF, and SIF, respectively.^10^, ^39^, ^40^ A detailed protocol for the simulated fluids is provided in a recent study.^41^ To promote efficiency of digestion (hydrolysis), appropriate and necessary conditions including shaking, temperature and control of digestion time are used for the different phases.^42^ Although studies have confirmed the potential of IGD to release BAPs from food samples,^38^, ^43^, ^44^ in some of these studies, the samples were also subjected to other forms of processing (e.g., fermentation), which could have influenced the effectiveness or otherwise of IGD in releasing BAPs from the sample. Therefore, it may be difficult to attribute the observed bioactivity solely to the IGD. For instance, fermented pea seeds were subjected to in IGD and the samples evaluated for ACE inhibitory potential,^45^ the authors found out that only the in vitro‐digested samples had ACE inhibition. In another study evaluating the impact of IGD on peptide profile and bioactivity of cooked and non‐cooked oat protein concentrates, the ability of IGD to produce peptides was very apparent as the digestates had significantly higher antioxidant properties when compared to the non‐digested samples.^46^

### 2.3 Production of bioactive peptides by fermentation

Fermentation of cereals and other grains is a renowned processing practice which is employed in Africa and Asia for the production of various fermented foods.^47^ Though not commonly employed like enzymes in the hydrolysis of protein for the production of BAPs, protein hydrolysates could also be obtained through the process of fermentation by the action of proteases released by the microorganisms during fermentation^48^ as has been shown in other foods. For instance, it has been reported that several potent BAPs with antioxidative, antihypertensive, antimicrobial, immuno‐modulating, anti‐inflammatory, and anti‐mutagenic properties were isolated from fermented milks.^49^ Microbial fermentation is a feasible alternative to enzymatic hydrolysis because it is more environmental friendly, cheaper and could be up‐scaled for industrial production of BAPs.^50^ During this period, the mixed fermentation occurring from the activities of successive microorganisms results in the decrease of pH and hence, the acidity of the medium.^47^ Starter cultures could also be employed for controlled and faster fermentation process whereby the sample is inoculated with microbial strain of interest. Regarding this, some of the microorganisms that have been used include different strains of lactic acid bacteria (LAB) (*Lactobacillus plantarum*, *Lb. rhamnosus*, *Lactococcus lactis*, and *Lactobacillus zeae*), *Bifidobacteria* and fungi (*Aspergillus oryzae*).^51^ Compared to other microorganism, LAB are preferred and often employed in fermentation for the production of BAPs because of efficiency of proteolysis, adaptability to different substrates and safety.^52^ Different methodological approaches had been reported in the production of hydrolysates through fermentation process. In most cases, the fermentation broth is centrifuged with or without inactivating the microorganism by heating the broth at about 100°C. Thereafter, the supernatant is collected directly as the hydrolysate^53^ or filtered with membrane filter to obtain the hydrolysate which may be lyophilized to extend its storability.^54^ In other cases, the fermented samples are still subjected to further enzymatic hydrolysis.^53^ Studies where flour rather than the grain were fermented or inoculated with starter cultures have also been reported. For instance, lupin, quinoa, and wheat flours were each fermented by *Bifidobacterium animalis*, *B. longum*, and *B. breve* and tested for antioxidant, antihypertensive, and cytotoxicity.^34^ Chickpeas were also subjected to solid‐state fermentation for 72 h and the aqueous extracted protein tested for radical scavenging capacity. The protein extracts showed increasing radical scavenging abilities as fermentation period progressed up till the 48 h and had maximum radical scavenging abilities of 86% and 85% for DPPH and OH assays, respectively.^55^ Expectedly, time and temperature have significant impacts on the protein and the peptide contents of fermented samples and these will influence their bioactivities. In a previous study where the peas were subjected to varying conditions of fermentation—temperature (22, 30, and 37°C) and time (3 h, 3 and 7 days), it was noted that the protein content for each of the temperature group decreased as fermentation period increased. In addition, only fermentation for 3 h increased the protein content beyond that of the control sample and protein content was maximum at 22°C.^53^ Conversely, the peptide contents, compared to the control, increased after 3 h, then decreased after 3 days and increased again after 7 days for fermentation at 22 and 30°C with the maximum peptide content obtained at 30°C after 7 days of fermentation while fermentation at 37°C resulted in progressive decrease in peptide contents throughout the fermentation period. The initial increase in peptide content is apparently due to the proteolytic activities of proteases released by the fermenting organisms. Since peptides/free amino acid residues are also required as source of nitrogen for microbial growth, especially oligopeptides, further reduction in peptide contents as fermentation progressed may be adduced to their utilization by the fermenting microorganism for growth. The observed increase in peptide content thereafter may be due to production of peptides not suitable as a nitrogen source for the microorganisms.^56^, ^57^ On the contrary,^58^ who evaluated the effect of fermentation time (12, 24, 36, 48, 60, and 72 h) on the peptide content of defatted wheat germ reported continual increase in peptide content till the 48 h before gradual decrease sets in. Therefore, the protein type and microorganisms involved may influence the degree of hydrolysis and hence the peptide content. The relationship between peptide contents and bioactivities has not always been directly correlated.

### 2.4 Production of bioactive peptide by malting/germination

Malting is the controlled germination of seed under precise environmental parameters such as humidity and temperature. Several metabolic processes take place in grains because of sprouting during the malting process. Besides reduction in anti‐nutritional factors and enhancement in nutritional parameters such as improved protein and starch digestibility, increase in bioavailability of minerals and vitamins, germination of grains has been reported to improve antioxidative and antihypertensive properties.^59^, ^60^ As would be expected, peptides are produced during germination as a result of the activities of proteolytic enzymes that are mobilized during the process and are involved in the breakdown of the protein. Hydrolysate from quinoa seed germinated for 0, 24, and 72 h and fermented with two different *Lactobacillus* strains were reported to have increased alpha glucosidase and ACE inhibitory properties though each strain had different inhibitory potentials in the two assays. Apparently, these effects were due to the combined impact of germination and fermentation. Mamilla and Mishra^59^ evaluated the impact of germination temperature (30 and 40°C) on the ACE inhibitory potentials of peptide extracts from some selected legumes (chickpea, soybean, red lentil, mung bean and kidney bean). They found out that increasing germination temperature to 40°C improved the ACE inhibitory potentials of all the samples compared to germination at 30°C except for mung bean and kidney bean. It is expected that enzyme activities will be more pronounced at higher temperature of 40°C compared to 30°C. Therefore, the observed increase in potency in some of the legumes may be due to further liberation of potent ACE peptides from the polypeptide while the reduction observed in mung bean and kidney bean may be due to dilution effect as a result of more hydrolysis. This may particularly be true considering the fact that at 30°C, mung bean and kidney bean already had considerably higher potency of 82 and 72 whereas chickpea, soybean and red lentils had 51%, 66%, and 49% ACE inhibition, respectively. Piñuel et al.^61^ also studied the effects of germination and hydrolysis on the antioxidant properties of protein isolate and hydrolysates from white, red, and black quinoa seeds. The authors discovered that the non‐hydrolysed proteins isolates from all the samples showed no radical scavenging ability against DPPH radicals. While the pepsin‐derived and pepsin‐pancreatin‐derived hydrolysates were able to scavenge DPPH radicals, hydrolysate from the non‐germinated seeds had better DPPH radical scavenging ability when compared to the germinated samples. Again, as previously noted, pepsin‐derived hydrolysate had lower potency in all the samples compared to the pepsin‐pancreatin digested sample. On the contrary, the non‐hydrolysed protein isolates had the highest ABTS radical scavenging abilities for all the samples. While this may confirm that different amino acid sequence are required for different bioactivities, it is difficult to determine the specific role of germination and further enzymatic hydrolysis in the observed bioactivities.

## 3 PURIFICATION, IDENTIFICATION, AND CHARACTERIZATION OF BIOACTIVE PEPTIDES

After protein hydrolysis, an essential step in obtaining a BAP is further fractionation of the protein in order to obtain a more purified peptide. Unpurified protein, that is, protein that are still intact and within the matrices of food are less active.^17^ This may be due to the interactions with other adhering components within the food, hence the need to isolate and purify protein. While varied results have been obtained, isolation of protein and further purification processes often lead to the production of peptides that are more potent when compared to the un‐isolated or unpurified parent protein. The potency, in most cases, increases progressively from the parent protein to isolate to hydrolysate and to peptide fractions. Apart from the removal of other adhering components (lipid, carbohydrate, etc.) from the protein, plausible explanations for the improved potency are the release of active amino acids from other peptide residue, reduced molecular weight, changes in net charges and formation of new amino acid sequences.^62^ Commonly used fractionation methods are membrane fractionation (e.g., ultrafiltration), size exclusion chromatography (e.g., gel filtration and ion exchange) and sodium dodecyl‐sulfate polyacrylamide gel electrophoresis (SDS PAGE). Generally, the purification process starts with ultrafiltration, then the most active peptides, based on some pre‐determined factors such as antioxidant or antihypertensive properties, is subjected to further purification by gel filtration.

The most active fractions from gel filtration may also be further purified in subsequent purification steps. Some of the purification techniques applied to grain proteins include ultrafiltration, gel filtration, and reverse phase high performance liquid chromatography (RP‐HPLC).^17^, ^25^, ^63^ In ultrafiltration, a solution of the protein hydrolysate is passed through membrane filters with specific molecular weight cut off (MWCO) usually between 1 and 10 kDa. Use of gel filtration chromatography, RP‐HPLC or reverse phase ultra‐fast liquid chromatography (RP‐UFLC) for further purification of grain proteins have also been reported.^17^ Finally, the sequence of amino acids in the BAP are identified using mass spectrometry techniques. In the study by Agrawal et al.,^17^ three different peptide purification methods were sequentially employed. Each stage of purification produced peptides that were more potent when compared to the parent/precursor protein. The result from the ultrafiltration techniques, where the smallest peptide (<3 kDa) showed highest potency also confirms previous observation that smaller peptides are move active than the bigger ones.^17^, ^64^ A similar trend was also obtained for foxtail millet prolamin^32^ where a similar purification process was employed. However, the <1 kDa fractions of the alcalase‐derived peptide was more active. Purification of the in vitro gastro‐intestinal simulated digest of four samples (raw, germinated, boiled, and microwaved) from foxtail millet protein by RP‐HPLC^25^ yielded other four sub‐fractions where the fourth fraction of all the samples showed significantly better antioxidative and anti‐inflammatory activities when compared to other sub‐fractions. However, these fractions had reduced metal ion chelating potential when compared to the ultra‐filtrated fractions. This may be due to dilution effect resulting from further fragmentation of the parent peptide. While some authors have reported reduction in metal ion chelating ability of fractions from RP‐HPLC others have also observed improved activity.^32^

Apparently, after hydrolysis and the various purification steps, it could be expected that the resulting peptides will possess different characteristics and features which would impact their bioactivities. In order to obtain necessary information which may help to understand, explain and predict bioactivities of peptides, it is often necessary to carry out the characterization of the peptides. A number of analytical protocols, from as simple as gel electrophoresis to the more complex spectrometry/chromatography, are available to evaluate the different physico‐chemical properties of the peptides. Various attributes of peptides which affect their bioactivities include, size, amino acid composition, the sequence or arrangement of amino acids, hydrophobicity, and charge. With respect to size, the degree of hydrolysis, which gives an indication of the activities and effectiveness of the hydrolyzing enzyme in breaking the peptide bonds, is an essential parameter providing some useful information related to the size of the peptides.^65^, ^66^ The molecular weight distribution, which gives a more quantitative estimate of the average sizes of the peptides can be determined using SDS PAGE, membrane filtration, size exclusion chromatography or gel filtration and fast protein liquid chromatography (FPLC).^65^ The structural features such as the amino acid composition and the sequences are determined using chromatographic or spectrometry systems. In fact, various types of chromatography have found different applications in the purification and characterization of BAPs. Notable among these are high performance liquid chromatography (HPLC) which is commonly used for determination of amino acid composition. Characterization of other specific feature of peptides can also be determined using RF‐HPLC or UF‐HPLC (hydrophobicity) and ion exchange chromatography (charge).^67^ Mass spectrometry techniques with some coupling accessories such as electrospray ionization (ESI), matrix‐assisted laser desorption/ionization time‐of‐flight/time‐of‐flight mass spectrometry (MALDI TOF/TOF MS),^66^ triple‐quadrupole MS and quadrupole time of flight (Q‐TOF) are prominent equipment of choice for the identification of peptide sequence as well as their quantitation.^68^ Peptides are identified and characterized by comparing experimental data from mass spectrometer with those already validated in sequence databases.^69^

Though there are still contrasting reports on the impact of peptide size on bioactivity, possibly due to other factors, such as the sequence of the amino acid, most of the reports have correlated improved bioactivity with higher degree of hydrolysis or small size (low‐molecular weight) peptides.

## 4 HEALTH PROMOTING PROPERTIES OF BIOACTIVE PEPTIDES FROM CEREAL AND LEGUME GRAIN

**FIGURE 1:**
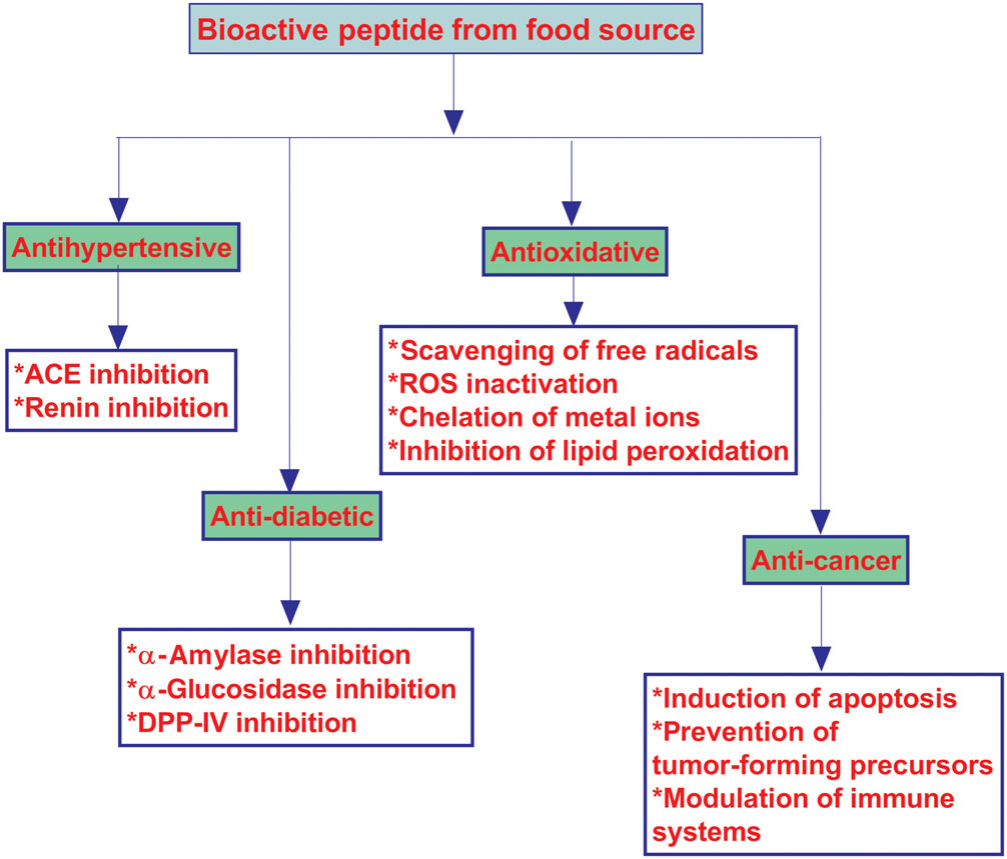
Schematic representation of some biofunctional activities of bioactive peptides

Studies ranging from in vitro, in vivo, and clinical trials have demonstrated the health promoting abilities of grain‐derived BAPs. These include antioxidant, anti‐inflammatory, antihypertensive, antidiabetic, anti‐cancer properties, and among others. A schematic representation of some selected bio‐functional (health promoting) properties of peptides is shown in Figure 1.

### 4.1 Antioxidant properties

Excessive production of free radicals has been implicated in the damage to essential macromolecules such as lipids and proteins including DNA. Oxidative stress occurs when the generation of free radicals such as reactive oxygen species exceed the potential of available antioxidants to control them, thus leading to the development of several degenerative diseases including cancer.^18^, ^70^ The ability of peptides to act as antioxidants and so prevent the buildup of free radicals and subsequent oxidative damage has been shown in several in vitro and in vivo studies.^8^, ^17^, ^32^, ^71^, ^72^ The proposed mechanisms by which BAPs exert antioxidant activity are by donation of free electrons, scavenging of free radicals or acting as metal chelators.^32^ hydrolyzed foxtail millet protein (prolamin) with alcalase and trypsin and evaluated their antioxidant (DPPH and ORAC) activities. The alcalase‐derived hydrolysate showed better antioxidative activities in the two assays when compared to the trypsin hydrolysate. This may be due to smaller peptides obtained from alcalase hydrolysis when compared to the trypsin‐hydrolysate counterpart more so that the degree of hydrolysis for alcalase was reported to be higher than that of trypsin.^32^

An IGD utilizing pepsin and pancreatin in a stepwise manner was carried out on foxtail millet protein.^25^ In the study, no significant differences were conferred on the bioactivities of the hydrolysates with respect to different pretreatment processes (germination, boiling, and microwaving). In fact, the hydrolysate from the raw sample had better metal ion chelation (MC) and FRAP abilities when compared to the pretreated samples. However, with further purification (ultrafiltration), some differences were observed with different fractions being active in different assays. For instance, the <3 kDa fraction from all the samples had better MC activities compared to other fractions. There was no specific trend for the ORAC and ABTS while all the >10 kDa fractions showed better ferric reducing antioxidant power. The <3 kDa (raw) also showed higher ability to inhibit nitric oxide production while the <3 kDa from the germinated and microwaved samples both showed significantly higher abilities to prevent albumin denaturation.

Ortiz‐Martinez et al.^73^ reported on the impact of protein type (albumin, globulin, prolamin, and glutelin), variety (normal maize, NM, and quality protein maize, QPM) and processing (hydrolysis: alcalase) on the antioxidative (oxygen radical capacity, ORAC) properties of the proteins. While hydrolysis generally increased the antioxidative potential of all the proteins for the two varieties, except for QPM glutelin hydrolysates, the degree of improvement in antioxidative potential among the proteins after hydrolysis differed significantly. For instance, hydrolysis of albumin and globulin fractions from the normal maize resulted in four‐fold increase in their ORAC values but three‐fold increase for their QPM hydrolysate counterparts. In addition, while the NM prolamin hydrolysate had seven‐fold increase (the highest) over the NM, the QPM prolamin hydrolysate on the other hand only had a two‐fold increase. The glutelin fractions however showed no statistically significant difference between the normal protein and the hydrolysates. In contrast, glutelin fractions from chickpeas (germinated in the presence of selenium) was reported to show higher cellular antioxidant ability when compared to the albumin fractions.^74^ Again, in the study on lupin, quinoa and wheat,^34^ fermentation increased the antioxidative (DPPH and ABTS) potential of the fermented flour hydrolysate significantly when compared to the control sample except for the wheat sample where there was only marginal increase for all the *Bifidobacteria* spp. used. The study also indicated there was no direct relationship with the duration of fermentation (0, 24, 48, and 72 h) and antioxidative potentials both for DPPH and ABTS assays except for lupin where increased fermentation period resulted in increased antioxidative potentials. Apparently, the different processing techniques employed and the nature of the native protein will lead to production of BAPs with different amino acid residues and hence, different bioactivities even when the same enzyme is used for hydrolysis.

However, while peptides' bioactivity has to do with the protein, there seems to be no direct correlation between bioactivities and protein contents or the protein yield. In the study on antioxidant characteristics of peptides from sorghum, where a series of enzymes were employed for hydrolysis, it was reported that everlase, one of the enzymes with lowest yield had better radical scavenging (DPPH) ability when compared to other enzymes with better recovery yield.^75^ Enzyme type and the degree of hydrolysis may also play significant roles in the potency of peptides since the duration of hydrolysis may determine size of peptides produced. This view is supported by a previous study on amaranth hydrolyzed with *Lactobacillus helveticus* and *Lactobacillus plantarum*.^35^ The 48‐h hydrolysis period showed that albumin and 11S‐globulin fractions hydrolyzed with *L. helveticus* had better ORAC at 8 and 14 h of hydrolysis whereas, the same samples had their best hydroxyl radical (OH^•^) scavenging abilities at 14 and 24 h of hydrolysis. It is also interesting to note that while albumin had overall higher potency in ORAC assay, the 11S‐globulin was the better sample for the OH^•^ assay which may suggest that different mechanisms are involved in scavenging free radicals and consequently different amino acid sequences are required. The impact of different hydrolyzing enzymes on the antioxidative properties of some selected grains are shown in Table 1. Previous studies^76^, ^77^ have reported the importance of the arrangement of amino acid in peptides relative to the C‐terminus. Since one of the mechanisms of an antioxidant in scavenging free radical is its ability to donate hydrogen, it has been observed that the presence of aromatic amino acids such as tyrosine at the C‐terminus of peptides confers higher radical scavenging ability on the peptide because of their hydroxyl group which can easily donate its hydrogen.^76^, ^77^ Other pretreatment techniques that have been applied to improve antioxidative property of peptide is electron beam irradiation.^78^ The authors, reported an increasing antioxidative (DPPH and ABTS) ability of the pea with increasing irradiation doze compared to non‐irradiated (control sample). The observed increase in the antioxidative properties of the pea was attributed to the unfolding of the protein by the electron beam‐generated energy, thereby exposing more of the peptide bonds to proteolytic effects of the hydrolyzing enzymes and thereby liberating more hydrophobic amino acids residues buried in the core of the protein. It is therefore not surprising that the authors reported highest antioxidant activity at the highest surface hydrophobicity obtained at the maximum irradiation dose used in the study. Hydrophobic amino acids are reported to be particularly important in preventing lipid peroxidation because they offer a better environment for the interaction between the peptide and other hydrophobic reactants such as fatty acids.^79^

### 4.2 Anti‐inflammatory/anticancer properties

Studies on BAPs, including those from grains have indicated their potential anti‐inflammatory and anti‐cancer properties including potential to prevent cancerous cells from proliferating. With little or no toxicity, unlike the conventional means of treatment, BAPs may become a potent alternative in prevention and treatment of cancers. Ayyash et al.^34^ evaluated the cytotoxicity of peptides from fermented grains (lupin, quinoa, and wheat) against colon (Caco‐2) and breast (MCF‐7) carcinoma cell lines. Only hydrolysates from lupin showed considerable inhibition against the two cancer cell lines and increased with prolonged fermentation (11%–79%). Peptide fractions isolated from quality protein maize showed significant anti‐proliferative properties against liver cancer—hepatocarcinoma human (HepG2) cells. Specifically, the most active fractions (F10) of the two genotypes (Asgrow‐773 and CML‐502) showed 95% and 85% anti‐proliferative abilities, respectively.^26^ The authors also reported at least, a four‐fold increase of the potential of the fractions in causing apoptosis in HepG2 when compared to the control. While the peptide sequences of the two genotypes were different, the range of hydrophobicity of the peptides were comparably similar.

Purified peptide from rice bran was demonstrated to exhibit 80%, 80%, 85%, and 69% inhibition at 1 mg/ml against human colon (Caco‐2), breast (MCF‐7), liver (HepG2), and lung (A‐549) cancer cell lines, respectively.^80^ Induction of apoptosis, prevention of tumor‐forming precursors and modulation of immune systems are some of the reported mechanisms for the anti‐proliferative activities of BAPs.^34^, ^81^ Several studies have reported cytotoxicity of peptide fractions from food sources against some cancer cell line, hence confirming the cytotoxicity ability.

### 4.3 Antihypertensive properties

**FIGURE 2:**
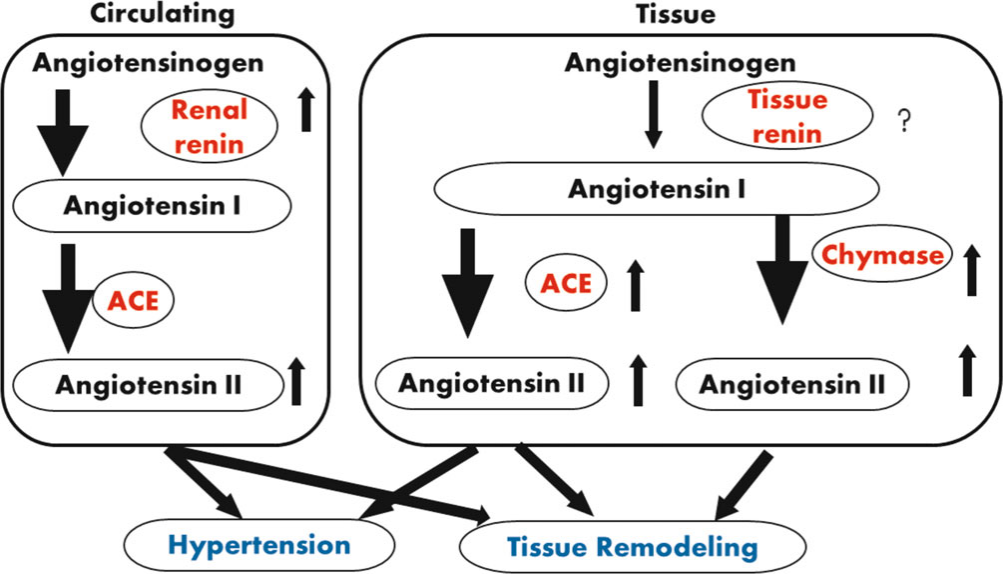
Renin‐angiotensin system. *Source*: Ref. 87

Globally, high blood pressure remains one of the major debilitating diseases affecting the populace and resulting in other fatal complications such as stroke, arteriosclerosis, ischemic heart disease, etc.^82^, ^83^ One of the promising means of combating this challenge with little or no side effects is through diet. A number of research studies have shown the ability of plant bioactive such as peptides in inhibiting angiotensin‐converting enzyme (ACE).^8^, ^84^, ^85^, ^86^ The ACE is implicated in two major pathways in the renin‐angiotensin‐aldosterone system (RAAS) which is responsible for the control of mammalian blood pressure. First, it converts the inactive angiotensin I to angiotensin II, a potent vasoconstrictor and secondly, it breaks down the arterial‐dilator peptidic hormone (bradykinin). Consequently, higher pressure is required for blood flow because of the constriction of the blood vessels (Figure 2).^82^, ^87^ Therefore, inhibition of ACE is one of the major mechanisms by which BAP control high‐blood pressure. Other reported means include inhibition of renin, stimulation of nitric oxide production, and by blocking angiotensin II receptors.^88^ Synthetic ACE inhibitory drugs are available for the management of hypertension but are often too expensive and present myriads side effects.^18^, ^89^ Potent ACE‐inhibitory peptides have been isolated from several natural sources (plant and animal) including grains.^18^

Peptides have demonstrated significantly promising potentials to inhibit ACE and hence, control or manage high‐blood pressure. Specifically, peptides with higher contents of hydrophobic amino acid residues in addition to low‐molecular weight, which promotes ease of absorption in the intestinal tract, are, reported to possess potent ACE‐inhibitory ability.^90^ Reports have shown that short peptides containing not more than nine amino acids residues are strong ACE inhibitors, especially, the di‐ and tri‐peptides because they are easily absorbed without further degradation. The position of individual amino acids relative to the C‐ or N‐terminal in the peptide sequence is also observed to play a significant role in the inhibitory activity against ACE. In this regard, the presence of tyrosine at the C‐terminus was reported to cause a slower but prolonged reduction in systolic blood pressure compared to the presence of phenylalanine which caused a faster but shorter period of activity.^79^ A study on the impact of foxtail millet protein hydrolysate in lowering blood pressure in spontaneously hypertensive rats^83^ showed that the peptide significantly lowered blood pressure at 200 mg/kg body weight. The authors also noted the impact of processing on the bioactivities of the peptides. They observed that the raw and extruded samples were more potent when compared to the fermented samples.

The impact of fermentation on ACE‐inhibitory potential of protein hydrolysates obtained from lupin, quinoa and wheat flours using *B. animalis* was also not significantly different from the control sample. However, the hydrolysates obtained from the same samples using *B. breve* and *B. longum* showed significantly higher potency when compared to the control sample.^34^ Contrary to this, germination of some selected legumes (chickpea, soybean, red lentil, mung bean, and kidney bean) at 30°C resulted in significantly improved ACE inhibition potential except in red lentil. Specifically, germination produced 72%, 99%, 225%, and 126% increases in ACE inhibition for chickpea, soybeans, mung bean, and kidney bean, respectively while red lentil had 25% reduction when compared to the non‐germinated samples.^59^ The importance of optimizing pretreatment/preprocessing conditions could also be noted from the study since each legume showed different ACE inhibition as the germination temperature changed. In this regard, as the grains were germinated at higher temperature (40°C), further 30% increase was obtained for chickpea and soybeans, respectively, 62% for red lentil while mung bean and kidney bean had 63% and 25% reduction, respectively over what was obtained at 30°C. Though all the grains had increased degree of hydrolysis at 40°C except kidney bean this reflected accordingly in their ACE inhibitory potential. However, mung bean with the highest degree of hydrolysis (at 40°C) had reduced ACE inhibition. This may confirm an earlier observation, though on renin, that the nature of protein and the position of the amino acid are more important than the size of the peptide.

Similarly, all protein (albumin, globulin 7S, globulin 11S, prolamin, and glutelin) hydrolysates obtained from millet grains subjected to 65 and 100°C heat treatment showed improved ACE inhibition when compared to the control sample and the hydrolysates from 65°C preheated grains showed overall higher potency.^91^ Though the degree of hydrolysis among the samples was relatively comparable, the in vitro potential bioavailability of the peptides for the 65°C treated sample was higher than others and this may be responsible for the overall improved ACE activity. Potent ACE‐inhibitory peptides were also obtained from pigeon pea. The pea hydrolyzed with pepsin, pancreatin, and pepsin‐pancreatin produced hydrolysates with over 70% inhibition against ACE. Evaluation of the blood pressure lowering potential of the pea peptides in spontaneously hypertensive rats showed that while the pepsin‐derived hydrolysate offered a quicker reduction in blood pressure within 2 h of administration, the pepsin‐pancreatin‐derived hydrolysate showed the best overall blood pressure reduction for the 24 h post‐administration period.^18^ The authors attributed the fast action of the pepsin‐derived hydrolysate to the fact that the protein had been predigested by the same enzyme (pepsin) which is involved in gastro‐intestinal digestion of proteins and therefore needed no further digestion in the intestine of the rat, hence the rapid action. Furthermore, the amino acid profile reported in the study showed that while the pepsin hydrolysates had higher hydrophobic amino acid content, the pepsin‐pancreatin hydrolysate was slightly higher in aromatic amino acids, specifically in tryptophan content and this may be responsible for the observed trend.

Likewise, Doyen^92^ obtained two cationic fractions (KCl‐F1 and KCl‐F2) from flaxseed protein hydrolysate by electrodialysis using ultrafiltration membranes which showed fast antihypertensive effect. They reported that the KCl‐F1 fraction and the final pea hydrolysate (FPH) significantly lowered systolic blood pressure (SBP) at 2, 4, 6, and 8 h after administration. The samples showed fast SBP lowering potential which was better than the antihypertensive drug (Captopril) used as the positive control 2 h post‐administration. These samples also sustained high potency comparable to that of the positive control after 4 and 6 h. According to the authors, while the antihypertensive property of the KCl‐F1 fraction could be due to its smaller peptides sizes, that of the FPH may be due to the concentration of relevant peptides with antihypertensive property by the electrodialysis with ultrafiltration membranes. With respect to the amino acid composition however, the FPH showed higher contents of hydrophobic and aromatic amino acids while the KC‐F1 had higher contents of histidine, lysine, and arginine. Apart from its ability in enhancing immune functions, the role of arginine in the formation of nitric oxide (a potent vasodilator) for the control of high‐blood pressure is well reported.^93^, ^94^ Lysine is also an essential amino acid which complements arginine. It is especially important in the catabolism of arginine. Therefore, a balanced arginine: lysine ratio is important and their regulatory effects on high‐blood pressure as well as cholesterol has been the focus of several studies.^95^, ^96^

Antihypertensive properties of protein and peptide fractions from some selected grains is shown in Table 2. As earlier reported,^75^ each enzyme is specific and will produce peptides with different amino acid residues than when another enzyme is used. This could positively or negatively impact the bioactivity of the peptide, depending on the assay type. Several previous studies where different enzymes were used for hydrolysis support this. For instance, hydrolysis of lima bean (*Phaseolus lunatus*) and Jamapa bean (*P. vulgaris*) seed with alcalase and flavourzyme produced hydrolysate with contrasting ACE inhibitory potential. While alcalase‐derived hydrolysate showed higher ACE inhibitory potential for *P. vulgaris*, the flavourzyme‐derived hydrolysate was the more potent for *P. lunatus*.^97^

**TABLE 2:** Antihypertensive properties of cereal and legume grain protein and peptide fractions including their amino acid sequences

Source	Processing, purification, and characterization method	Test	Result	Sequence	Reference
Lima bean (*Phaseolus lunatus*) and Jamapa bean (*Phaseolus vulgaris*)	Protein isolation: isoelectric precipitation	ACE IC_50_ (mg/ml)	–	–	^97^
	Protein hydrolysis: Alcalase (ALH and AJH) and flavourzyme (FLH and FJH)		ALH:0.056 AJH: 0.061 FLH:0.007 FLH:0.127		
	Fractionation/purification: Ultrafiltration (1, 3, 5, and 10 kDa)		FLH: 17.5 (>10 kDa) AJH: 63.8 (<1 kDa)		
Pigeon pea	Protein isolation: Isoelectric precipitation (PpI)	In vitro (ACE and renin, %) and In vivo in SHR (mm Hg)	ACE PpI: 62 PpH: 77 PaH: 75 PpH:77 Renin PpI: – PpH: 08 PaH: 12 PpH:10 In vivo 2 h: −31 (PpH) 4 h: −33 (PpH) 6 h: −35 (PaH) 8 h: −35 (PaH) 12 h: −38 (PpH) 24 h: −32 (PpH)	–	^18^
	Protein hydrolysis: Pepsin (PpH), Pancreatin (PaH), pepsin‐pancreatin (PpH) Sample concentration ACE: 0.5 mg/ml Renin: 1 mg/ml SHR: 100 mg/kg BW				
Quinoa	Pretreatment Germination at 0, 24, and 72 h	–	QLCZ 0 h: 80 24 h: 85 72 h: 84 QLCSY13 0 h: 85 24 h: 85 72 h: 83	QLCZ RGAIVL GVRGRGRIV GGRFA QLCSY13 LGGIWHL VAHPVF IRAMPVAV ALFPTHR LAHMIVAGA	^98^
	Protein hydrolysis Fermentation with *Lactobacillus casei* (QLCZ) and *L. casei* SY13 (QLCSY13)	ACE (%) at 400 ul/ml			
	Peptide Identification: RP‐HPLC–MS/MS				
α‐Kafirin (Sorghum)	Protein isolation: Alcoholic (tert–butanol) extraction	–	Fraction F38: 1.3	–	^99^
	Protein hydrolysis: Chymotrypsin	–			
	Fractionation/purification: Gel filtration (with fraction 38 being the most active)	ACE IC_50_ (ug/ml)			

Abbreviations: ACE, angiotensin converting enzyme; paH, pigeon pes pancreatin hydrolysate; PpH, pigeon pea pepsin hydrolysate; PpI, pigeon pea protein isolate; RP‐HPLC, reverse phase high performance liquid chromatography; SHR, spontaneously hypertensive rat.

### 4.4 Antidiabetic properties

Diabetes has been described as a chronic metabolic disorder resulting from the inability of the body to either produce or use insulin for the control of postprandial hyperglycemia. Just like other diseases such as hypertension, synthetic antidiabetic drugs such as glibenclamide and metformin are available for diabetes treatment but with other undesirable side effects. Consequently, studies are establishing the potential of food‐derived peptides in the treatment or control of diabetes.^92^, ^100^ Most of these studies aim to control diabetes by delaying or preventing sudden rise in hyperglycemia through inhibition of alpha amylase, alpha glucosidase, and dipeptidyl peptidase IV which are enzymes all involved in metabolism of carbohydrate at the different stages. Furthermore, peptides have been reported to play other significant roles in the control of hyperglycemia such as encouraging insulin secretion, promoting glucose absorption in peripheral tissue or decreasing its absorption in the gut.^101^

The in‐vitro antidiabetic (alpha amylase and alpha glucosidase inhibition) reported for protein from fermented lupin, quinoa, and wheat showed that grain hydrolysates possessed antidiabetic properties.^34^ The fermented grain hydrolysates were comparably effective against the two enzymes except for wheat where reduced inhibition was observed. Furthermore, the study showed that fermentation for 24 h produced hydrolysates with significantly higher inhibition when compared to the control sample. Since the degree of hydrolysis also increased significantly from 24 h of fermentation, the improved antidiabetic properties obtained in the study may be due to the smaller size of the peptides. Also, peptide fraction (KCl‐F1) obtained through electrodialysis with ultrafiltration membrane from flaxseed protein hydrolysate was reported to possess antidiabetic property through its ability to increase glucose update in L6 skeletal muscle cells both in basal (without insulin) and insulin‐stimulated conditions.^92^ Valencia‐Mejía et al.^101^ also reported the antidiabetic potential of easy‐to‐cook (ETC) and hard‐to‐cook (HTC) common beans (*P. vulgaris*). The beans protein isolate as well as the hydrolysates and fractions were evaluated for alpha amylase and alpha glucosidase inhibition. Generally, all fractions from both ETC and HTC beans had reduced alpha amylase and alpha glucosidase inhibitory potentials after hydrolysis. However, fractions from the non‐hydrolysed and those from ETC and HTC bean hydrolysates showed higher inhibition against alpha glucosidase (36%–89%) than alpha amylase (3%–89%) though the ETC slightly had higher overall alpha glucosidase inhibition. It was also noted that the smallest fractions (<3) in all the samples had significantly higher inhibition against both enzymes when compared to other fractions (3–10 and >10 kDa), indicating the role of size in peptides' bioactivity. Further in vivo study of the various fractions revealed that fractions from the non‐hydrolysed sample and specifically, the <3 kDa, had similar antihyperglycemic effect as the control sample (acarbose). Unlike the in vitro assay however where hydrolysis led to reduced bioactivity, the <3 kDa fractions from ETC and HTC bean hydrolysates were reported to show higher antihyperglycemic effects than acarbose.

Protein hydrolysates from millet grains preheated at 65 and 100°C^91^ showed considerable inhibition against alpha amylase and alpha glucosidase activities though most of the hydrolysates obtained from the sample pretreated at 65°C had no inhibitory properties against alpha amylase. In general, the peptides, including those from the control samples were more active against alpha glucosidase than alpha amylase. The study also revealed that different proteins were active at the different pre‐treatment temperature. For instance, in alpha glucosidase inhibition assay, globulin 11S was the most active for the control sample and also the sample preheated at 100°C while at 65°C, prolamin was the most active protein. Interestingly, the glutelin fractions which showed no activities for the control sample had considerable alpha‐glucosidase inhibition after preheating. Preheating may have been responsible for the release of potent peptides with alpha glucosidase inhibitory ability.^91^ BAPs from rice and rice bran protein hydrolysates showed no inhibition against alpha glucosidase but both had Ic_50_ of 1.45 and 1.28 mg/ml potencies, respectively against DPP‐IV which was significantly higher than 27.55 mg/ml obtained for sake lees hydrolysate.^27^ Evaluation of alpha glucosidase inhibition by peptides from sprouted quinoa yoghurt showed moderate dose‐dependent potency of over 40% at the minimum concentration of 50 ul/ml. At higher concentration of 400 ul/ml, most of the samples at the different germination regimes had significantly higher anti alpha glucosidase inhibitory properties of over 80%.^98^ While the obtained values in the study were slightly lower than the positive control antidiabetic drug (Acarbose) with 82%–93% potency range, nevertheless, the results showed the potentials of grain‐derived peptides as antidiabetic agent. Table 3 shows the antidiabetic properties of protein and peptide fractions from some selected grains.

**TABLE 3:** Antidiabetic properties of cereal and legume grain protein and peptide fractions including their amino acid sequences

Source	Processing, purification, and characterization method	Test	Remark	Sequence	Reference
Rice protein (Oryza protein‐P70)	Protein hydrolysis: Denazyme Fractionation/purification: gel filtration chromatography Peptide identification: LC–MS	α‐glucosidase and DPP‐IV (IC_50_ mg/ml)	No inhibition against α‐amylase; DPP‐IV: 1.45	Leu‐Pro Ile‐Pro Met‐Pro Val‐Pro	^27^
Rice bran	Protein isolation: Alkaline extraction Protein hydrolysis: Umamizyme Fractionation/purification: gel filtration chromatography Peptide identification: HPLC, LC–MS	DPP‐IV (IC_50_ mg/ml)	Umamizyme G:2.3 Bioprase: 26.3	Leu‐Pro Ile‐Pro (Umamizyme)	^102^
Easy and hard to cook common beans (*Phaseolus vulgaris*)	Protein isolation: Alkaline extraction	In vitro antidiabetic assays using α‐amylase and α‐glucosidase In vivo studies using male Wistar rats	The hydrolyzed and non‐hydrolyzed protein were ultra‐filtrated and the <3 kDa fraction was the most active in all the samples. The non‐hydrolyzed ETC and HTC had 32, 89, 89, and 87 while the hydrolysates had 53, 54, 17, and 34 for AA and AG, respectively at 10/mg/ml sample concentration. In vivo studies, <3 kDa from non‐hydrolyzed sample had better anti‐hyperglycemic effect compared to the HTC counter parts while hydrolysates from both fractions of ETC and HTC significantly higher anti‐hyperglycemic effects than acarbose at 5 mg/ml sample concentration	–	^101^
	Protein hydrolysis: gastrointestinal digestion (sequential hydrolysis by pepsin and pancreatin)				
	Fractionation/purification: Ultrafiltration (3 and 10 kDa MWCO membranes)				
Soybean	Germination (6 days)	–	–	DPP‐IV inhibitory peptide *From F1* NNDDRDS VVNPDNNEN LSSTEAQQS NAENNQRN IKSQSES EEPQQPQQ GQSSRPQD LAGNQEQE NLKSQQ QEPQESQQ SQRPQDRHQ QQQQQGGSQSQ QQQQQGGSQSQKG PETMQQQQQQ SDESTESETEQA From F2 RNLQGENEEEDSGA VTRGQGKV KKGVIT IMSDESTESETEQA From F3 NALKPDNRIESEGG SSPDIYNPQAGSVT NALKPDNRIESEGG RQNIGQNSSPDIYNPQAG NALKPDNRIESEGG VVAEQAGEQGFE HKNKNPF	^103^
	Protein isolation: Alkaline extraction followed by isoelectric precipitation	–	–		
	Enzymatic hydrolysis: gastrointestinal digestion (sequential hydrolysis by pepsin and pancreatin)	DPP‐IV α‐Amylase α‐Glucosidase determined as maltase and sucrose activities (IC_50_, mg/ml)	DPP‐IV: 1.49 α‐Amylase: 1.7 Maltase activity: 3.73 Sucrose activity: 2.90		
	Fractionation/purification: Ultrafiltration RP‐HPLC (5–10 kDa) 1 mg/ml sample concentration Peptide identification: RP‐HPLC–MS/MS	DPP‐IV α‐Amylase & α‐Glucosidase determined as maltase and sucrose activities (IC_50_, mg/ml)	DPP‐IV: 0.91 (5–10 kDa) α‐Amylase: 4.8 (>10 kDa) Maltase activity: 2.56 (<5 kDa) Sucrose activity: 1.23 (<5 kDa)		
		DPP‐IV (IC_50_, mg/ml) α‐Amylase (%) α‐Glucosidase (%) determined as maltase and sucrose activities	DPP‐IV: 0.81 (F3) α‐Amylase: 85 (F1) Maltase activity: 33 (F4) Sucrose activity: 21 (F1, F2, and F3)		
Corn germ	Protein hydrolysis: enzymatic hydrolysis with alcalase, flavourzyme, and trypsin	α‐Amylase (10 mg/ml), α‐glucosidase (20 mg/ml) and DPP‐IV (5 mg/ml) (all in %)	α‐Amylase: 54 α‐glucosidase: 15 DPP‐IV: 38	–	^30^
	Fractionation/purification: Ultrafiltration (2, 10 and 100 kDa MWCO membranes) RP‐HPLC		α‐Amylase: 61 (2–10 kDa) α‐glucosidase: 42 (2–10 kDa) DPP‐IV: 56 (<2 kDa) α‐amylase: 71 (F2, alcalase) α‐glucosidase: 41 (F1, flavourzyme) DPP‐IV: 56 (F1, alcalase)		
Lupin, quinoa, wheat	Protein hydrolysis: Solid state fermentation with three strains of *bifidobacteria* (*B. animalis*, *B. breve*, and *B. longum*) fermented for 0, 24, 48, and 72 h	α‐Amylase and α‐glucosidase (%)	α‐Amylase Lupin: 72 (*B. animalis*, 72 h) Quinoa: 60 (*B. longum*, 0 h) Wheat: 30 (*B. longum*, 0 h) α‐glucosidase Lupin: 70 (*B. breve*, 72 h) Quinoa: 72 (*B. breve*, 48 h) Wheat: 65 (*B. breve*, 48 h)	–	^34^
Quinoa	Pretreatment Germination at 0, 24, and 72 h	–	QLCZ 0 h: 77 24 h: 81 72 h: 85 QLCSY13 0 h: 81 24 h: 85 72 h: 87	QLCZ KSFGSSNI KDLQL MIKLRSTAKN QLCSY13 VAHPVF LAHMIVAGA KLTPQMA	^98^
	Protein hydrolysis Fermentation with *Lactobacillus casei* (QLCZ) and *L. casei* SY13 (QLCSY13) Fractionation/purification: RP‐HPLC Peptide identification: RP‐HPLC–MS/MS	α‐Glucosidase (%) 400 ul/ml			

Abbreviations: DPP‐IV, dipeptidyl peptidase IV; ETC, easy‐to‐cook; HTC, hard‐to‐cook; MWCO, molecular weight cut off; RP‐HPLC, reverse phase high performance liquid chromatography.

## 5 CONCLUSION

Research on BAPs from cereal and legume grains is still quite limited when compared to other food items and most of the research already carried out are done without identifying the sequence of the BAP or characterizing the constituent amino acids. However, studies on the antioxidative, anticancer/inflammatory, antihypertensive, and antidiabetic properties that have been reported show that there are promising prospects of obtaining potent BAPs from grains which could be utilized in the development of functional foods and nutraceuticals. As at the time of this review, data is lacking on the quantitative structure–activity relationship (QSAR) of BAPs from most grains as well as utilization and impact of novel food processing technologies (e.g., pulsed electric field and ultrasound‐assisted extraction) which are aimed at improving protein yield and/or bioactivities. Studies on BAPs potential of cereal and legume grains and in vivo evaluation of the peptide bioactivities will be important for their maximum exploitation in the development of functional foods and nutraceuticals.

## AUTHOR CONTRIBUTIONS

Kwaku Gyebi Duodu conceptualized the idea, Taiwo Ayodele Aderinola compiled and wrote the manuscript. Kwaku Gyebi Duodu reviewed and corrected the manuscript. Both authors approved the final manuscript submitted for publication.

## CONFLICT OF INTEREST

The authors declared no conflicts of interest.

**Abbreviations**
ABTS: 2,2′‐azino‐bis(3‐ethylbenzothiazoline‐6‐sulfonic acid)
ACE: angiotensin converting enzyme
BAPs: bioactive peptides
DPPH: 2,2‐diphenyl‐1‐picrylhydrazyl
DPP‐IV: dipeptidyl peptidase IV
ESI: electrospray ionization
ETC: easy‐to‐cook
FPH: final pea hydrolysate
FPLC: fast protein liquid chromatography
FRAP: ferric reducing antioxidant power
HPLC: high performance liquid chromatography
HTC: hard‐to‐cook
IGD: in vitro gastrointestinal digestion
MALDI TOF/TOF MS: matrix‐assisted laser desorption/ionization time‐of‐flight/time‐of‐flight mass spectrometry
MC: metal ion chelation
MWCO: molecular weight cut off
NM: normal maize
OH: hydroxyl radical
ORAC: oxygen radical absorbance capacity
paH: pigeon pes pancreatin hydrolysate
PHR: protein hydrolysate from raw sample
PHT: protein hydrolysate form boiled sample
PpaH: pigeon pea pepsin‐pancreatin hydrolysate
PpH: pigeon pea pepsin hydrolysate
PpI: pigeon pea protein isolate
PIT: protein isolation from raw sample
PIT: protein isolation from boiled sample
QPM: quality protein maize
QSAR: quantitative structure–activity relationship
Q‐TOF: quadrupole time of flight
RAAS: renin‐angiotensin‐aldosterone system
RP‐HPLC: reverse phase high performance liquid chromatography
RP‐UFLC: reverse phase ultra‐fast liquid chromatography
SBP: systolic blood pressure
SDS PAGE: sodium dodecyl‐sulfate polyacrylamide gel electrophoresis
SGF: simulated gastric fluid
SHR: spontaneously hypertensive rat
SIF: simulated intestinal fluid
SSF: simulated salivary fluid

## Data Availability

Data sharing not applicable ‐ no new data generated, or the article describes entirely theoretical research
